# Do you see the problem? Visualising a generalised ‘complex local system’ of antibiotic prescribing across the United Kingdom using qualitative interview data

**DOI:** 10.1080/09581596.2023.2210743

**Published:** 2023-06-13

**Authors:** Rebecca E. Glover, Nicholas B. Mays, Alec Fraser

**Affiliations:** a Policy Innovation Research Unit, London School of Hygiene and Tropical Medicine, London, UK; b Public Services Management and Organisation, Kings College London, London, UK

**Keywords:** Antimicrobial resistance, prescribing, complex system

## Abstract

Antimicrobial resistance (AMR) is often referred to as a complex problem embedded in a complex system. Despite this insight, interventions in AMR, and in particular in antibiotic prescribing, tend to be narrowly focused on the behaviour of individual prescribers using the tools of performance monitoring and management rather than attempting to bring about more systemic change. In this paper, we aim to elucidate the nature of the local antibiotic prescribing ‘system’ based on 71 semi-structured interviews undertaken in six local areas across the United Kingdom (UK). We applied complex systems theory and systems mapping methods to our qualitative data to deepen our understanding of the interactions among antibiotic prescribing interventions and the wider health system. We found that a complex and interacting set of proximal and distal factors can have unpredictable effects in different local systems in the UK. Ultimately, enacting performance management-based interventions in the absence of in-depth contextual understandings about other pressures prescribers face is a recipe for temporary solutions, waning intervention effectiveness, and unintended consequences. We hope our insights will enable policy makers and academics to devise and evaluate interventions in future in a manner that better reflects and responds to the dynamics of complex local prescribing systems.

## Background

### Antimicrobial resistance

Antimicrobial resistance (AMR) is a problem that is widely understood to require action from multiple actors at the individual, local, regional, national, and supranational levels to mitigate the emergence of resistance, and the transmission and burden of resultant infection. It is thus understood to be – and frequently described as – ‘complex’ (Graham et al., 2019; O’Neill, 2016; Wernli, Jørgensen, Harbarth, et al., 2017; Wernli, Jørgensen, Morel, et al., 2017). However, while governments, bureaucrats, and non-governmental organisations, among others, may adopt complexity narratives, AMR policy ‘solutions’ tend to be grounded in quick fixes, or ‘downstream’ interventions that target patient or prescriber individual behaviour (Chandler, 2019; Glover et al., 2022; Willis & Chandler, 2019). This tendency to focus on individual responsibility rather than attempting to influence wider systems has been critiqued in recent years (*Drug-Resistant Infections*, 2017).

This paper aims to further understandings of antibiotic prescribing behaviours in the United Kingdom (UK) at a local level using complex systems mapping. We are particularly interested in the states, interventions, or conditions that were reported to impact on prescribing (intentionally, or unintentionally). We conclude by suggesting how the adoption of local-level systems mapping can be applied to the AMR policy development process, or any policy development process, in order to visualise and understand the surrounding systems.

## Complex systems

Complex systems have been defined as ‘a collection of elements (e.g. subsystems, sectors) with interconnections between those elements, and other characteristics including feedback, non-linearity, adaptation, and emergence’ (Cavill et al., 2020). Complex systems tend to share certain characteristics, such as heterogeneity (diversity of actors, and diversity of their goals), interdependence of the different elements, emergence of unexpected phenomena (e.g. novel resistance genes), and ‘tipping points’ (small changes tipping the whole system out of a period of stability) (Hammond, 2009). Complex systems theory has been used across public health to critically appraise the level and scope of current health interventions (Knai et al., 2018; Petticrew et al., 2013). Conceptualising public health domains using complex systems theory has helped in many ways. First, to demonstrate the inappropriate nature both of blaming individuals for their choices when they are being acted upon by a complex system, and second, to demonstrate the inappropriate nature of downstream interventions when these exist without complementary structural ‘upstream’ interventions (Cavill et al., 2020).

Systems mapping can also provide more explicit options when it comes to selecting and targeting interventions. It is frequently helpful to visualise complex systems since such visualisations can provide greater confidence when acting upon the system, even if they are not complete (Knai et al., 2018). Visualising the system can also act as a reference point for future research, by identifying untested assumptions and known unknowns.

It is of course important to be aware of the use and misuse of complexity theory and the concept of a ‘complex problem’. Complexity can be a problematic and weaponised concept within health, especially when used by the corporate sector in order to resist interventions that would promote public health. Petticrew et al. (2017) argue that adopting a complexity lens can be an industry strategy to dismiss evidence that a product is harmful; or to lobby against population health measures. Additionally, Savona et al. (2020) describe how complexity discourse can act as a ‘rhetorical smokescreen’ for public health interventions in the field of obesity and be misused to argue for individual-level rather than structural change, which would benefit the Unhealthy Commodities Industries (UCIs). The UCIs use the existence of complexity to argue that prevailing systems are *too* complex to be modified in a meaningful way. By contrast, our intention when adopting a complex systems lens is to better understand the AMR policy ecosystem, and the consequences of developing certain AMR policies or interventions as opposed to others, hoping that such insights will enable policy makers and academics to devise and evaluate interventions in future that are better placed to alter the dynamics of complex local systems.

## Antibiotic prescribing as a complex system

Antibiotic prescribing, which is a behaviour associated with the exacerbation – and perhaps possible mitigation – of AMR, is itself considered to be complex because of the interconnectedness between community, primary and secondary care, the range of prescribers, and the complexity of patient pathways (Birgand et al., 2018; Lorencatto et al., 2018). The last 25 years have seen extensive efforts towards antibiotic prescribing change. While antibiotic prescribing used to be limited typically to doctors and dentists, it is now increasingly shared among doctors, nurses, pharmacists, physiotherapists, midwives and others in the United Kingdom. The trend of widening antibiotic prescribing responsibilities has occurred especially in Western European and Anglo-Saxon high income countries. For example, Australia, Canada, Ireland, New Zealand, Sweden, the UK and the US have had nurse prescribing for over a decade. Pharmacist prescribers are less common, though exist in the UK, Canada, and New Zealand.

The widening range of professionals able to prescribe, and the implications of this, have attracted the attention of medical sociologists, in particular, Broom. Broom et al. (2014, 2015) empirical work describes and elucidates the knowledges, practices and sense-making behaviours of Australian hospital doctors, and hospital pharmacists. In the UK, many more types of health professionals can prescribe antibiotics than in Australia, so prescribing may require additional interprofessional negotiation and discretion. However, in England, policy makers’ approach to improving the use of antibiotics has been largely ‘top-down’, or led from the centre, hierarchical, and governed by disciplinary and financial individually-and hospital/practice-focused measures (Borek et al., 2020). Behaviour modification interventions (including nudge interventions), economic incentives for ‘appropriate’ prescribing, monitoring and evaluation interventions, and other similar performance management interventions continue to be deployed (Allison et al., 2020; Bou-Antoun et al., 2018). This includes: GPs and hospital doctors being monitored by antimicrobial pharmacists and local and national commissioning bodies or equivalent medicines management officials in the rest of the UK; the national performance indicators being collected and collated; and other nudge interventions such as the Chief Medical Officer (CMO) writing to high prescribing GP practices explaining how much higher their prescribing was than that of their peers (Gilchrist et al., 2015).

## Local complex systems

Top-down interventions interact with one another at the local level. Orton et al. (2017 describe how a systems lens can be applied to a local empowerment intervention to reduce health inequalities, but as yet remains under-theorised. Durie and Wyatt (2013) used complexity theory to advance community health programming, and insist that a complex adaptive systems theoretical lens should be applied to the local level because the quality of past and present relationships between local actors can govern behaviours and outcomes far more than any intervention’s constituent components.

The UK health systems are not more or less complex than any others; and in many ways the choice of complex systems theory to analyse the qualitative data in this paper is not in response to the systems’ characteristics, but in response to how the field of AMR has developed and focused on magic bullets and downstream technological innovation. We argue that it may be time to change the way we tackle antibiotic prescribing interventions; adopting a complexity science lens builds in *ex ante* the notion of unintended consequences and interventions pulling in opposite directions, and brings to the fore the notion that these interventions will necessarily interact within the system. Of course, undertaking this research in this way may consequently put into the background the very real and important concepts of public administration, professional culture, and organisational management; however as noted above, this is where the predominant evidence base is well-developed; we wanted to try another way of situating, and rendering visual, the AMR problem.

## Methods

### Study design

The data for the current analysis were collected as part of a policy evaluation for the Department of Health and Social Care in the United Kingdom on the implementation of the UK Five-Year Antimicrobial Resistance Strategy 2013–18. One part of the study comprised six local health system case studies across the United Kingdom. Case studies are useful tools for understanding local level interactions, and local context (Fraser, 2020).

We selected six clinical commissioning groups (CCGs) (or equivalent outside England) as local study sites because prescribing guidance was typically set at this level. CCGs, now replaced by integrated care boards, were extant from 2012–2022 in England, and were responsible for commissioning many primary and secondary healthcare services.

We obtained host institution ethical approval (LSHTM 14396) and Health Research Authority (project 220612) approval.

## Data collection

### Case study selection

Our six case study sites are now in the public domain, so we will name our sites here, and then refer only to the professional’s designation without reference to their case study site (these were Betsi Cadwaladr, Blackburn with Darwen, Camden, Western Health and Social Care, Greater Glasgow and Clyde, and West Norfolk). The sites were selected to include the maximum variation of factors that may plausibly affect AMR rates, as reported in the literature on AMR transmission: urban/rural, affluent/deprived, high/low HCAI rates, and high/low rates of antibiotic prescribing, all of which have been posited to influence the emergence, transmission, and burden of AMR (Curtis et al., 2019). Selection of unique sites in diverse contexts provided greater opportunities for learning.

### Qualitative interviews

We conducted between 10 and 14 semi-structured interviews in each local case study site between January 2017 and September 2018, a total of 71 interviews. The interviews varied in length between 15 and 90 minutes. We sampled our professionals purposively (Ames et al., 2019). We aimed to include from each case study site: microbiologists; infectious disease consultants; junior doctors; nurse prescribers; ward nurses; consultant pharmacists; antimicrobial pharmacists; medicines management teams; chief executives of commissioning and planning bodies and acute trusts; professional education deliverers; GPs; public health officials; and infection prevention and control experts. Among the completed 71 interviews, at least two of each type of professional was interviewed. Professionals were interviewed using a topic guide that was co-constructed by the five researchers who conducted the interviews, and covered a range of topics including: the UK’s five-year antimicrobial resistance strategy; patient care; logistics of bed management; resourcing; electronic prescribing; recruitment and retention of specialists and staffing more broadly; guidance on antimicrobial therapies; quality premiums and incentives; and audit and monitoring of antibiotic prescribing.

### Data analysis

**Figure 1.**
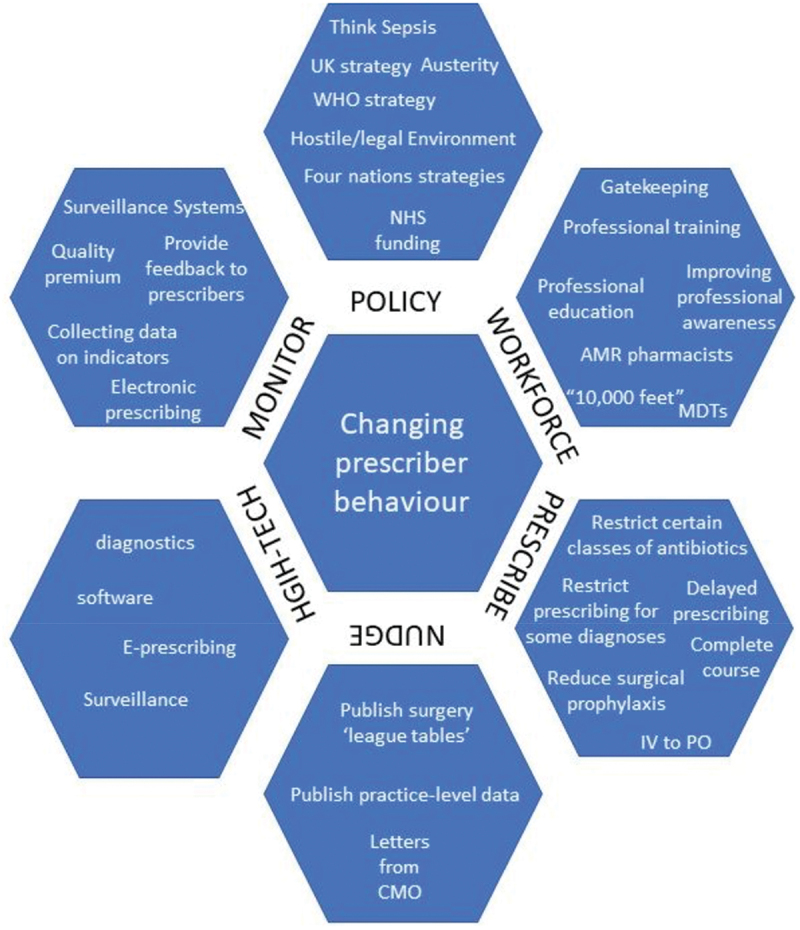
The six domains that thematically arose from the qualitative data and example phrases, policies, and topics from within each domain.

**Figure 2.**
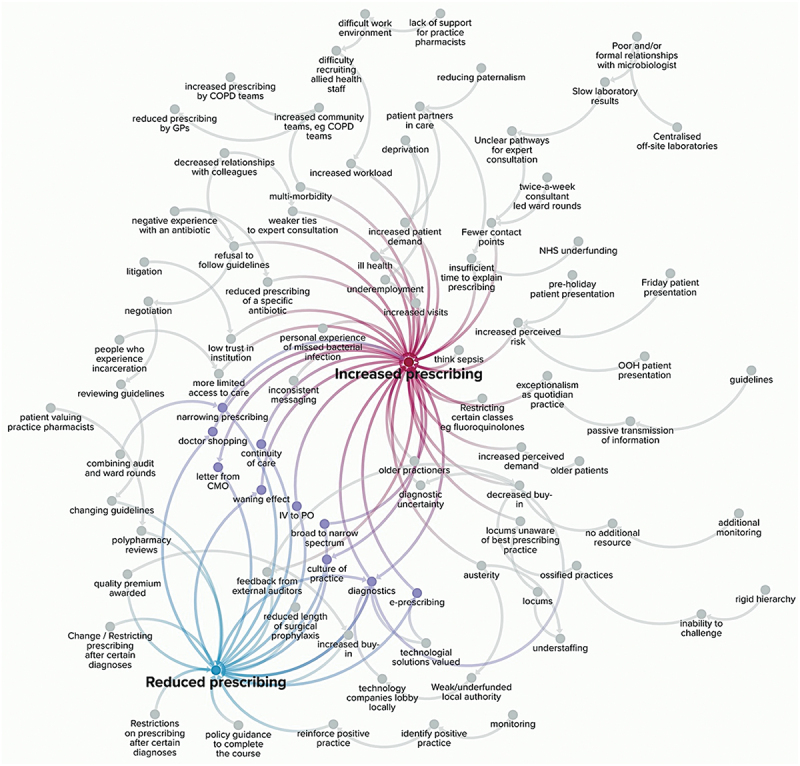
Generalised local complex antibiotic prescribing system, aggregated from all 71 qualitative interviews across the six case study sites.

This was a three-part analytical process. We first inductively coded prescribers’ references to conditions that impacted prescribing, and grouped them thematically. These are summarised in Figure 1. Second, led by complex systems theory, we posited that it would be possible to capture the reported linkages between the conditions described within the six intervention domains in Figure 1 and reported prescribing decisions – that is to say, we were sensitised to areas where our interviewees reported that a condition, such as a financial incentive, led to them increasing or decreasing their antibiotic prescribing. When an interviewee reported a clear connection, we coded this in Excel, aggregated all six case study sites, then entered these data into KUMU.io, (Kumu, 2023) a software that makes relationship maps. Because our case studies were selected for maximum heterogeneity but not representativeness, we did not map the strength of the relationships. For example, if ‘guidelines’ were reported by ten respondents but ‘technology’ was reported by 50 respondents, we did not visually capture technology as five times more important than guidelines, because we were aiming to comment on the heterogeneity of concepts, not to quantitatively assess their relative importance. We mapped all the relationships reported, in Figure 2. Third, once we had mapped the system, we observed in Figure 2 that there were certain conditions that had been reported by our interviewees to lead to both increased and decreased prescribing. We returned to our empirical data in the richest three areas to elucidate these tensions. Throughout, we employed an interpretivist qualitative approach using both inductive and deductive logics (Corbin & Strauss, 2008). Whilst our interview data do not codify, reflect, or describe an exhaustive system, system maps are one way to represent the themes and decision pathways that emerged, and how policies relate to practice (Cavill et al., 2020). They are also a tool that allows for the capture of divergence through both positive and negative causal pathways.

## Results

### Inductive classification and linking reported cause and effect

Many interventions, actions, and policies were reported to impact on prescribing practices. These are aggregated in Figure 1, and grouped into six domains: policy, workforce, prescribing guidance, nudge interventions, high-tech interventions, and audit and monitoring.

Next we coded the relationships between conditions and effect on prescribing. Classical hierarchies of evidence might dispute qualitative research’s ability to disentangle cause and effect, but, informed by complex systems theory and qualitative methodologies, we can richly linger, and reflect, on *reported* cause and effect. That is to say, our interviewees told us, in detail, which interventions led to increased or decreased prescribing. Data from all six local case study sites are amalgamated into Figure 2. Each single entry in the system has been coded to a primary topic from Figure 1, and so that these themes are more visible, they have been highlighted in Figures S1–S6.

What is of particular note in our study is that our interviewees clearly delineate proximal and distal causes of higher and lower rates of antibiotic prescribing, not all of which would be classed as intentional AMR interventions. For example, in the ‘policy’ domain there were clearly delineated distal impacts on prescribing such as deprivation and NHS underfunding (Figure S1), whereas in the ‘monitoring’ domain, interventions were described as impacting the prescribing decisions more immediately (Figure S2). The types of intervention reported were stark reminders of the macro- meso- and micro-level factors (inter)acting in the complex AMR system. For example, there were local prescribing policies (Figure S3) such as restricting certain classes of antibiotic within a hospital to microbiologist-released only, and national-level nudge interventions (Figure S4), like sending letters from the Chief Medical Officer (CMO) to high-prescribing GP practices in England. Workforce issues permeated all six case study sites, and their impact was felt across the system in varied ways (Figure S5), whereas high-tech interventions, such as rapid diagnostics and electronic prescribing, are the most proximal interventions to the actual prescription decision moment, and also the most ambiguous in terms of reported direction and impact of prescribing (Figure S6). This is of particular note due to the continued top-down policy push to use more diagnostics across the UK in all care contexts (*Tackling Antimicrobial Resistance 2019–2024: The UK’s Five-Year National Action Plan*, 2019). In all six case study sites in the UK, austerity (ie the UK government’s regime of post-2008 spending cuts designed to balance the public finances) and their consequences, such as increasing health inequalities, were reported to increase prescribing in some distal capacity, such as through worsening quality of life, increasing multi-morbidities, and thereby increasing the number of episodes of illness requiring antibiotics.

**Figure 3.**
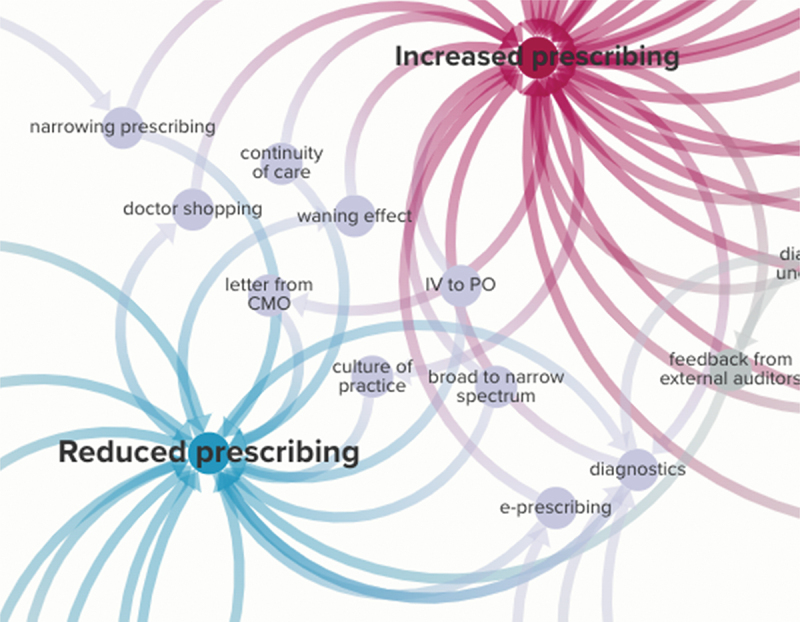
The conditions, policies, or interventions that led to reports of increased and decreased prescribing by our interviewees, over all six local case study sites.

Frequently, one interviewee’s experience with an intervention ran counter to the experience of another’s. In mapping the links between a condition or intervention and change in prescribing, we found chains of behaviours that reportedly led to increased prescribing, decreased prescribing, or both (Figure 3).

We paid particular attention to the themes that richly emerged out of many local sites, which were: a letter sent from the Chief Medical Officer (CMO) to high prescribing GP practices designed to nudge prescribers to lower their antibiotic prescribing by informing them that their prescribing was higher than their peers’; high tech interventions; and prescribing guidance(s). We reflect on these below to provide some sense of our empirical dataset.

**Nudge:**
the ‘ten percent letter’

The CMO sent a letter to GP practices that were considered high prescribers. This nudge intervention was mentioned across many of our interviews as leading to decreased prescribing in some contexts, and increased prescribing in others. One respondent, a doctor, said:

Now we’re getting these CMO letters, […] You know, ‘justify why you’re here’. Whereas we’ve done it in a much more subtly, you know, talking to them about why are you here, what can we do? How can we help you? We’ve always had that kind of approach and that’s helped an open culture, nobody feels like they’re being, you know, dictated to […] [The CMO letter] can undermine what we’re doing locally.

In the literature, the letter from the English CMO is often seen as a success (Ratajczak et al., 2019). It is low-cost, and studies have validated the effectiveness of using social norms letters to reduce prescribing (Hallsworth et al., 2016). Some critiques exist in the literature (Allison et al., 2020) and among our interviewees. Still, there seems to be policy consensus that it is useful, even though some interviewees in our study reported behavioural intervention fatigue which may reduce the effectiveness of this and similar interventions in the longer term.

**High-tech interventions:**
‘it is the worst idea’

Two types of technology were reported to lead to both increased and decreased prescribing: rapid diagnostic testing, and electronic prescribing, with the vast majority of the tension in this theme centred on diagnostic testing. In the UK, diagnostics for hospital laboratories are bought on a lab-by-lab basis, and there is no requirement to standardise the technologies available in different laboratories UK-wide. Therefore, there is an inherent tension between top-down government decrees and bottom-up decision-making at the local level. GP practices are free to purchase their own C-reactive protein (CRP) tests. But this cost, unless incentivised by local CCGs, or within the context of a pilot or trial, is borne by the surgery, and has consequently met with ambivalence (Bates et al., 2017; Van den Bruel et al., 2016).

A GP succinctly explained the conflict that they felt about CRP tests, which can often help to distinguish between bacterial and viral infections:

Interviewer: How do you feel about [CRP tests]?

Respondent: Mixed.

Interviewer: Okay, tell me about that.

Respondent: Okay, well it would be nice in a way to have the tests because it might help us to be reassured that we’re not missing anything serious, but my worry would be that if we had it people would find out about it and then they’d think, oh well I’ll just go and see this doctor and have the test and then I’ll know it’s nothing serious.

Ambivalence tended toward negativity in those who had experience of adoption of these tests. In one case study site, a rapid test for methicillin-resistant *Staphylococcus aureus*. (MRSA) had been adopted, but following a trial, the Trust decided to revert back to culture plates. A consultant explains:

Respondent: MRSA screening in this Trust – we used to do a PCR test which was a two-hour test. Actually if you were at [lab in case study region] it would take you at least a day to get that PCR test to the laboratory and it would probably be two days from the day you’re taking it that you get a result. So you advertise it as a two-hour test which was £32 but actually two days later you got the result.

Interviewer: So you went back to culture?

Respondent: Yes. We went back to culture, saved £1 million.

The sentiments about diagnostics also merged with the distal consequences of centralisation, and also called into question the role of the rapid diagnostic tests themselves.

A senior manager in a different Trust offered an assessment of laboratory centralisation plans: ‘It is the worst idea’. They continued, comparing current practice to the proposed plans: ‘My local lab downstairs, you know, five minutes’ walk away isn’t near enough […] What the hell are we doing trying to move it up the motorway?’ When asked who was pushing the plan onto senior managers, another senior manager said ‘well, the Department of Health are pushing it, for a start […]’. And when asked what national level initiatives would help them most with their job, the same senior manager said ‘not to do it’, referring to the laboratory centralisation.

Only one senior manager – the only one that was not clinically trained – expressed that the plan to centralise laboratories was a step forward, due largely to the money that the Department of Health had earmarked for the centralisation process. This senior manager stated that ‘the role of point of care testing is to allow and support greater centralisation of services’.

**Prescribing guidance:**
‘ … prevent them prescribing’

The competing influences of different sets of prescribing guidance emerged readily from our complexity map, including potential tensions between the intravenous to oral (IV to PO) antibiotic policies, broad-to-narrow spectrum policies, and front-line antibiotics vs antibiotics of last resort policies. Prescribing guidance is unusual within UK AMR, since it is largely locally situated, and can respond to local-level antibiotic resistance data. In one case study site, there was an initially unsuccessful introduction of new guidelines to encourage use of a broad-spectrum antibiotic called gentamycin for patients who were suspected of having serious bloodstream infections. In spite of the guidance being compiled by local consultants in infectious diseases, microbiology and pharmacy, the surgeons refused to prescribe gentamycin following the case of a patient developing hearing loss, a known side-effect of that drug. Instead, the surgeons prescribed meropenem, a broad-spectrum antibiotic of last resort. One infectious disease consultant described how prescribing advisers worked to limit the use of meropenem:

… so we introduced an antibiotic called aztreonam as an alternative to gentamycin, to try and prevent them prescribing […] meropenem. And, with a lot of very good work from our antimicrobial pharmacist and our local microbiologist in that particular hospital, they switched away from these very broad spectrum antibiotics, to these narrow spectrum antibiotics.

In this case, a third antibiotic was introduced as a negotiated ‘compromise’ antibiotic; it is clear that initial views on what constituted an ‘appropriate’ antibiotic prescription differed among professionals.

## Discussion

Our analysis of these 71 interviews highlights that there are strong contextual factors, such as austerity and other policies in the non-AMR policy ecosystem, that impact on antibiotic prescribing and that are not sufficiently recognised in the field. First, in the policy realm, reliance on near-patient and/or near-provider solutions such as nudge/behavioural interventions, or top-down, centralised command-and-control policy mandates, appears to be the norm. Limiting interventions in this area to monitoring and evaluation interventions, high-tech interventions, and behavioural nudges seems unlikely to match the impact of underfunding, austerity, the ill health of the populations being served, shorter primary care appointment times, UK-wide inequalities, and the many and varied ways in which the policy and workforce concerns permeated our systems maps. Second, even among narrow, proximal antibiotic interventions, such as rapid diagnostic testing and prescribing guidance, impacts differed depending on local complexities and context. The extent and scope of unintended and interacting interventions and contexts revealed in the interviews – including the impacts of wider systems – warrants urgent consideration in future research.

The systems maps we developed build on the difficulties of defining ‘appropriate prescribing’: we, as so many before us, were not able to directly examine this concept, so instead we have defaulted to examining increased and decreased prescribing, because the appropriateness of any particular antibiotic therapy is constructed amongst professionals, and professional culture, and changes over time. Consequently, appropriate prescribing for any given problem cannot readily be described in binary terms, or even as a spectrum between most and least appropriate, but rather as a constellation of managed uncertainties, or, in other words, a complex adaptive local system. The type and magnitude of these uncertainties influenced prescribers’ decisions as to whether, and when to prescribe antibiotics, and if so, which ones.

Departing from the context of Broom et al’s work, which is frequently set in Australia, where pharmacists could not prescribe antibiotics either in the community or in hospitals at the time of writing, our research in the UK occurs where many professionals are able to prescribe antibiotics. Care settings are important, and different enough to drive major differences in practice and antibiotic prescribing; the direction of health policies in the UK and other high income countries is toward increasing integration of services, and innovation in health care delivery (Briggs et al., 2020; Mounier-Jack et al., 2017). Current UK policies, for example, have encouraged GP practices to form clusters, to strengthen links between hospitals and community services, and to integrate health and social care, with varying degrees of success across the four nations (Baxter et al., 2018). Therefore, working to further develop an understanding of the complex antibiotic prescribing system across and among local care settings may support future AMR policy.

Our study clearly demonstrates that professionals cite a much wider range of topics as impacting their prescribing decisions than simply their medical training, AMR policies or interventions, or even patient expectations. Our interviewees clearly link prescribing practices to the structural drivers of AMR as a public health problem. We know from the literature that this is the case: pollution, poor quality homes, and an obesogenic environment create multimorbidities which lead to millions of antibiotic scripts each year (Maccioni et al., 2018). AMR is no different to any other aspect of public health or health inequalities in its political nature; as stressed by the sociologists Salway and Green (2017):

The lack of attention to wider societal processes, and predominant focus on individual ‘life-style’ behaviours, as causes of health inequalities in recent years is not simply a product of medical models of evidence generation. Rather, this epistemological stance has tended to coalesce with an ideological position that locates the roots of disadvantage with individual traits and diverts attention away from policy solutions that are unpalatable to those in powerful positions.

These causal links to wider societal and structural public health must be considered as at least partly underlying the demand for acute antibiotic scripts in the community. To implement AMR interventions with long-term impact, AMR should be joined up with health systems strengthening, poverty reduction policies, and a strong regulatory environment, as has been insisted upon at the global level (Livernash, 2019).

The consequences of intervening in a local system in a dominantly downstream way without adequately conceptualising and understanding a local system as complex is that there will be interactions between the intervention and the environment that may decrease or nullify the impact of, in this case, prescribing practices. Downstream behaviour change or nudge discourse in concert with a crisis narrative can have unanticipated effects. If there is a belief that downstream intervention will work – without acknowledging the importance of context – then there may be attempts to sequester funds to intervene in this area, and in particular, to intervene in downstream technological or pharmaceutical domains. And ultimately, presenting the complex system as a set of technical challenges that the technocratic-industrial AMR complex can respond to using audit and monitoring policies, and some downstream interventions, may reassure policymakers in the short-term, whilst removing their ability to meaningfully intervene in a sustainable and effective way (Grant & Hood, 2017).

## Conclusion

This study has shown that local actors identify unpredictable, divergent, and contrary effects of similar-seeming AMR-related prescribing interventions in different local systems in the UK. Also, this study suggests how decisions taken in the name of austerity can make it much, much harder to achieve long-term gains in AMR. The acknowledgment of local complexities in antibiotic prescribing neither abnegates the need for individual-level intervention, nor invalidates the (limited) benefits of downstream/nudge interventions. However, intervening in the local system in a downstream manner simply because it is presumed to be less complex than the national or international AMR system is a recipe for temporary solutions, waning intervention effectiveness, and unintended consequences caused by the interplay between such interventions and the system. Moreover, this is setting up prescribers for failure because it does not take account of the complex constellation of managed uncertainties that they negotiate. The findings in this paper lead us to query where public-health trained professionals fit into the local prescribing system. Accepting current time, funding, and capacity constraints, our analysis reveals the importance – at all levels within the prescribing system – to have access to population and public health experts to take into consideration systems, contexts, and the messiness – but ultimate worth – of upstream complex interventions.

## Supplementary Material

Supplemental Material

Supplemental Material

Supplemental Material

Supplemental Material

Supplemental Material

Supplemental Material
