# Supplementary figures and images for: Do you see the problem? Visualising a generalised ‘complex local system’ of antibiotic prescribing across the United Kingdom using qualitative interview data
Source: Crit Public Health. 2023 Jun 13;33(4):459–71. doi: 10.1080/09581596.2023.2210743 (PMC10388844; doi:10.1080/09581596.2023.2210743)

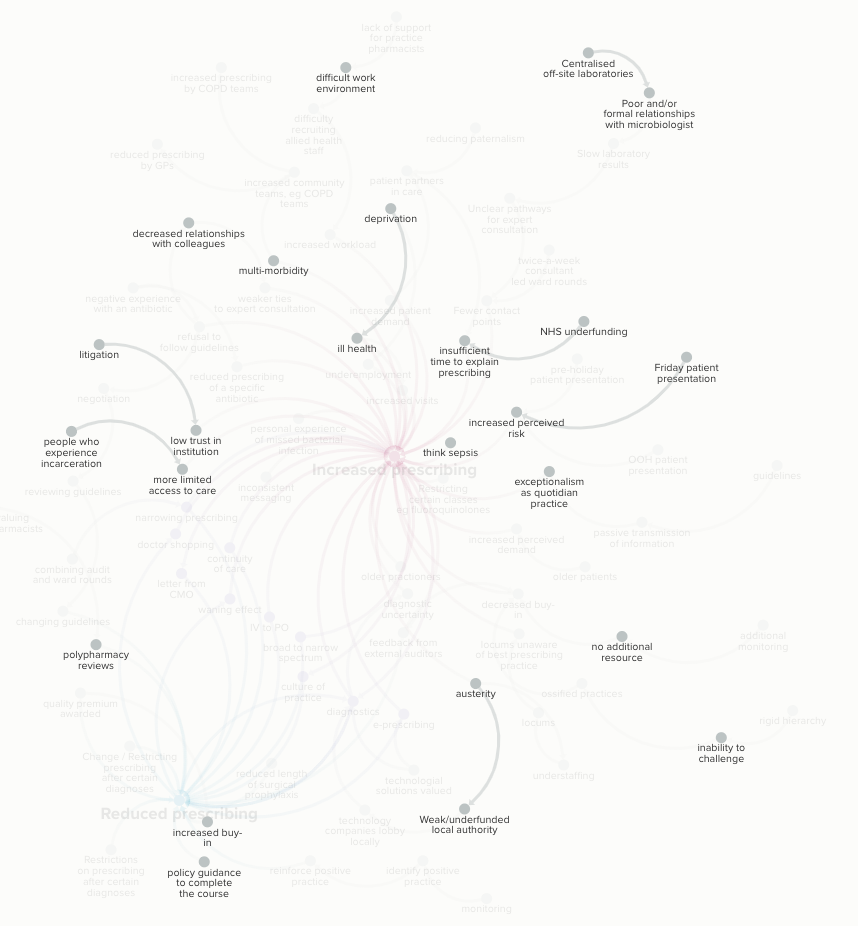

Supplement: Supplemental Material [file CCPH_A_2210743_SM0640.png]

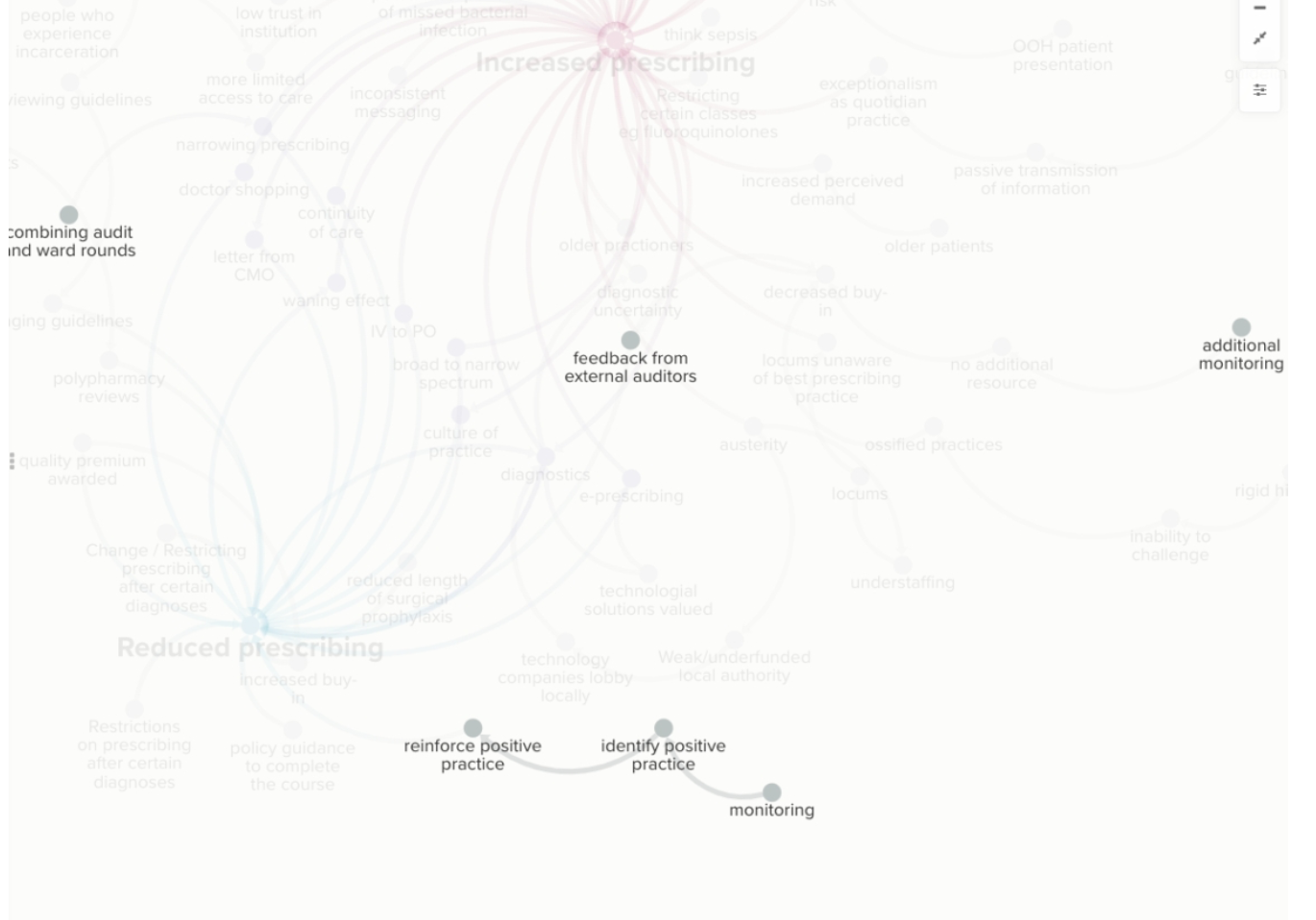

Supplement: Supplemental Material [file CCPH_A_2210743_SM0366.png]

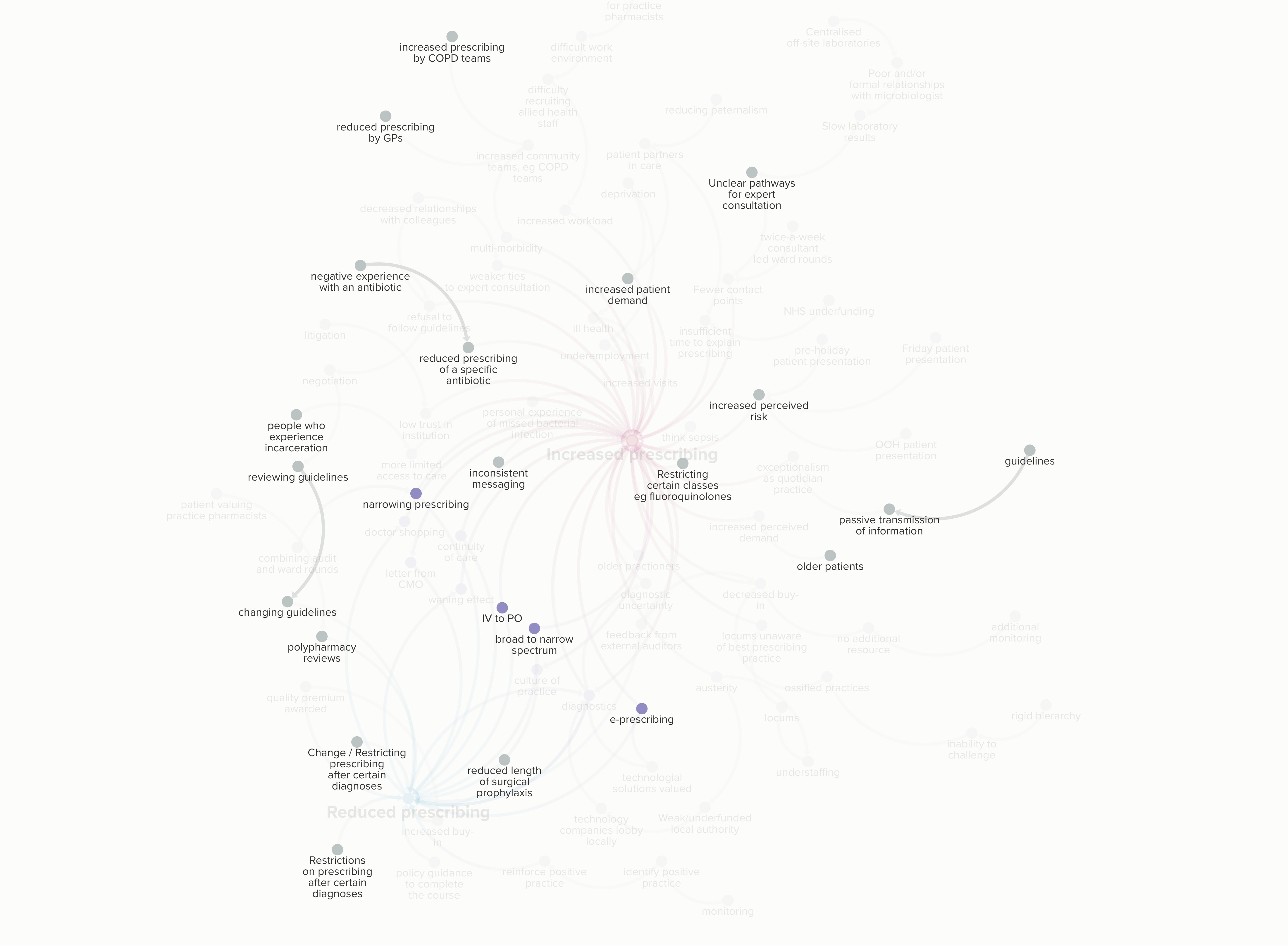

Supplement: Supplemental Material [file CCPH_A_2210743_SM0363.jpeg]

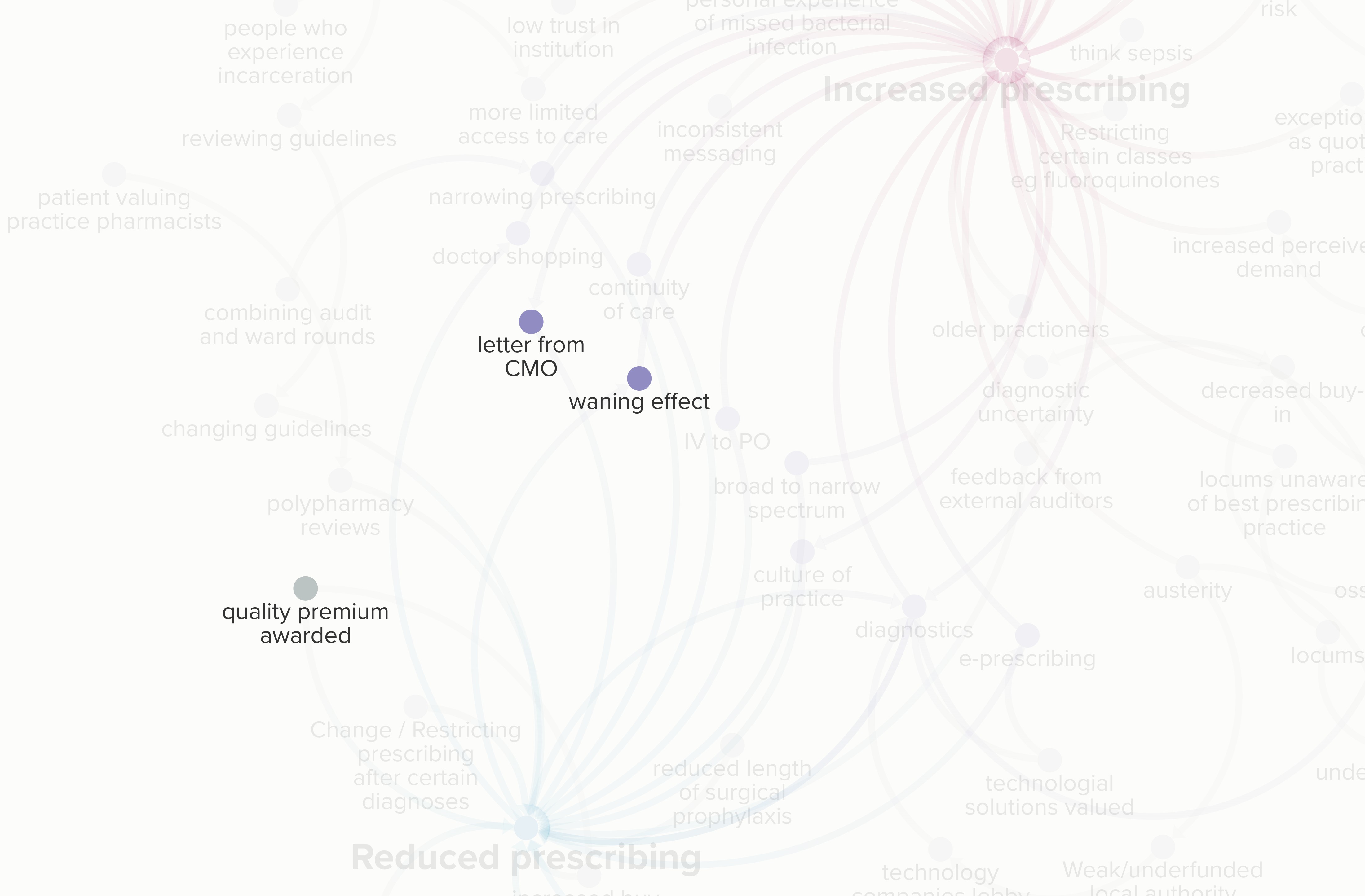

Supplement: Supplemental Material [file CCPH_A_2210743_SM0355.jpeg]

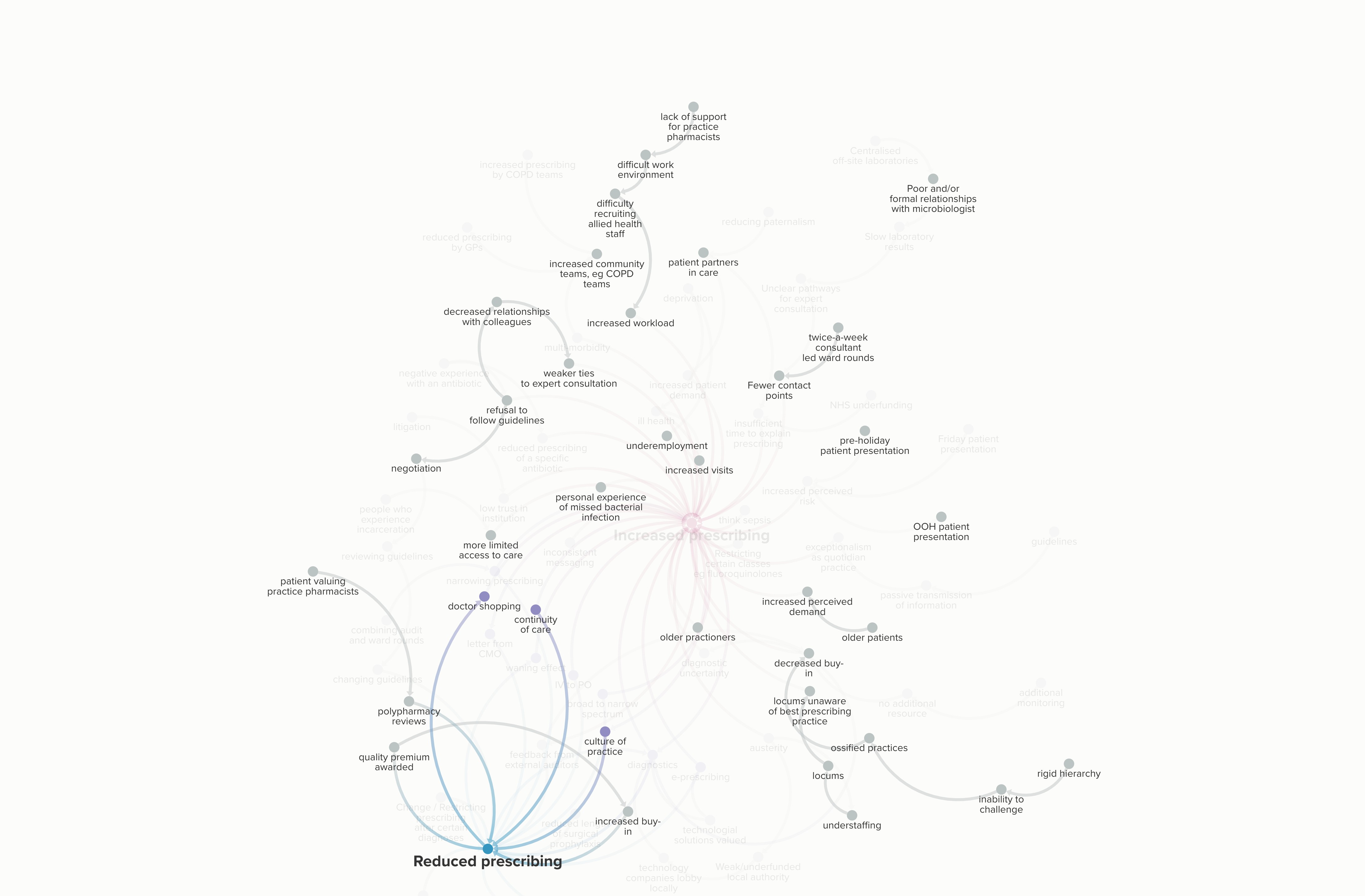

Supplement: Supplemental Material [file CCPH_A_2210743_SM0347.jpeg]

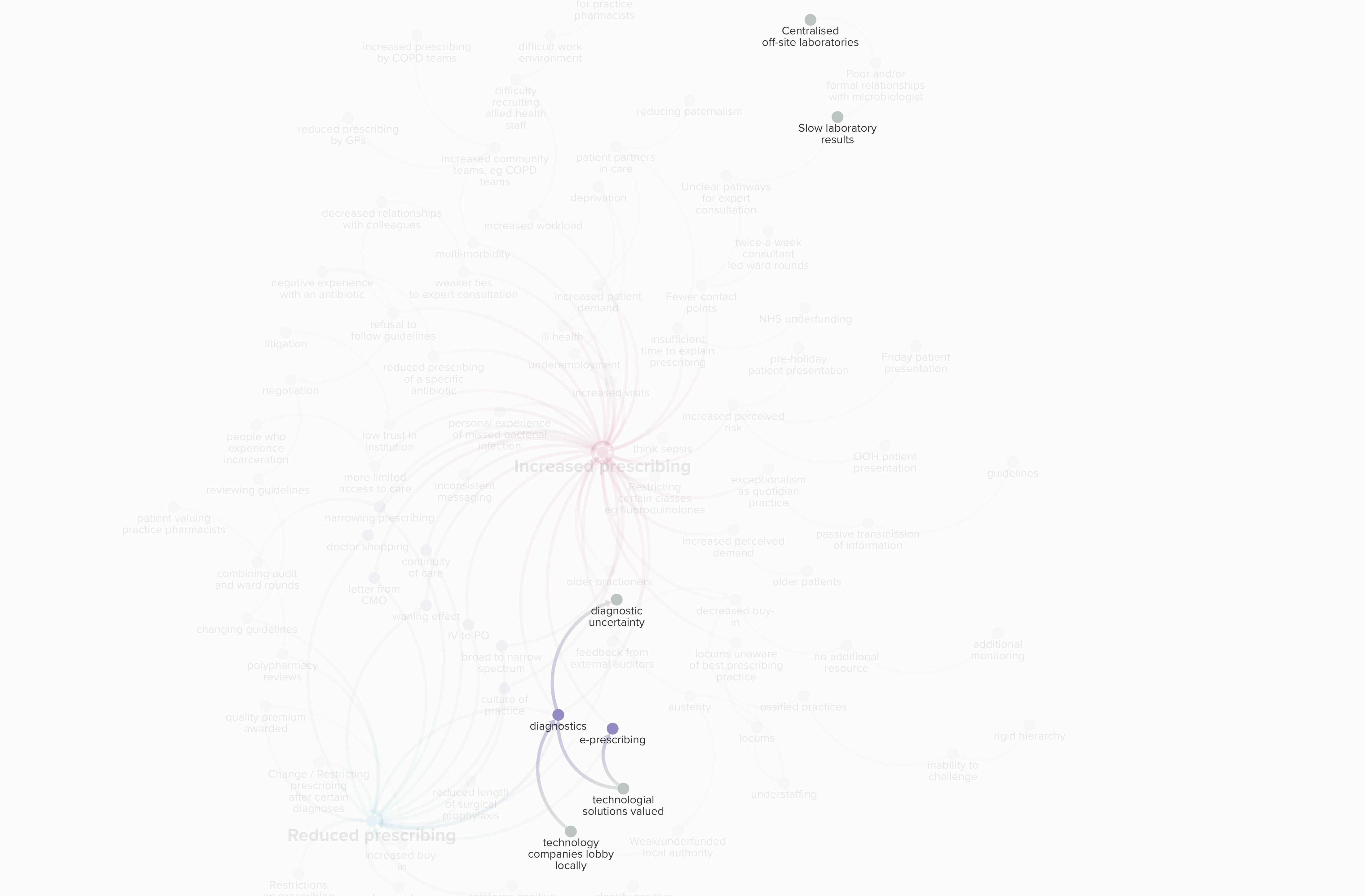

Supplement: Supplemental Material [file CCPH_A_2210743_SM0346.jpeg]
